# Reply to Dolu, K.O. Comment on “Topaloglu et al. Machine Learning-Driven Lung Sound Analysis: Novel Methodology for Asthma Diagnosis. *Adv. Respir. Med.* 2025, *93*, 32”

**DOI:** 10.3390/arm94020016

**Published:** 2026-02-28

**Authors:** Ihsan Topaloglu, Gulfem Ozduygu, Cagri Atasoy, Guntug Batıhan, Damla Serce, Gulsah Inanc, Mutlu Onur Güçsav, Arif Metehan Yıldız, Turker Tuncer, Sengul Dogan, Prabal Datta Barua

**Affiliations:** 1 Department of Pulmonology, Faculty of Medicine, Kafkas University, 36000 Kars, Turkey; 2 Department of Thoracic Surgery, Faculty of Medicine, Kafkas University, 36000 Kars, Turkey; 3 Department of Pulmonology, Faculty of Medicine, Health Sciences University, Izmir City Hospital, 35620 Izmir, Turkey; 4 Faculty of Medicine, Kafkas University, 36000 Kars, Turkey; 5 Department of Pulmonology, Faculty of Medicine, Bakırçay University, 35665 Izmir, Turkey; 6 Department of Computer Engineering, Faculty of Engineering, Ardahan University, 75000 Ardahan, Turkey; 7 Department of Digital Forensics Engineering, College of Technology, Firat University, 23119 Elazig, Turkey; 8 School of Business (Information System), University of Southern Queensland, Toowoomba, QLD 4350, Australia

Thank you for forwarding the external comment regarding our published article (Machine Learning-Driven Lung Sound Analysis: Novel Methodology for Asthma Diagnosis) [1]. We appreciate the opportunity to clarify the points raised by the commenter and provide additional methodological details for transparency.

**(1)**  **Clarification on the external validation dataset (ICBHI 2017)**

We would like to clarify that our external validation was not conducted on the full canonical ICBHI 2017 database containing multiple respiratory pathologies. Since the ICBHI 2017 dataset includes heterogeneous diagnostic categories, we manually selected only the recordings corresponding to the asthma and healthy control classes in order to establish a binary evaluation scenario consistent with our study design. This selection was performed strictly based on class labels (not based on signal quality or outcome).

Subsequently, the selected recordings were segmented into 3 s windows to match the preprocessing workflow applied to our primary dataset and to ensure methodological consistency. Therefore, the reported “ICBHI 2017” external validation results should be interpreted as results obtained from an ICBHI-derived asthma vs. healthy subset rather than the entire dataset.

**(2)**  **Apparent discrepancy between accuracy (Table 4) and ROC-AUC/AP (Figure 5)**

The commenter noted a potential inconsistency between the accuracy reported in Table 4 and the ROC-AUC/Average Precision (AP) values shown in Figure 5 for the Narrow Neural Network model. We would like to clarify that these metrics were derived from the same 10-fold cross-validation outputs but they do not measure the same property.

Specifically, the reported accuracy (99.63%) is a threshold-dependent metric computed using out-of-fold hard decision class labels at the selected decision threshold. In contrast, the ROC-AUC (0.877) and AP (0.868) are threshold-independent metrics computed from out-of-fold continuous model scores/probabilities across all possible thresholds, reflecting ranking/separation capability rather than a single-threshold classification outcome. A Narrow Neural Network architecture (shallower/lower-parameter structure) may achieve high accuracy at a specific threshold while producing less calibrated or less separable score distributions, leading to comparatively lower AUC/AP values. Therefore, this observation does not indicate a contradiction, but rather reflects the different nature of hard-label accuracy versus score-based discrimination metrics.

**(3)**  **Cross-validation, segmentation, and leakage concerns**

We acknowledge the importance of avoiding data leakage in segmentation-based respiratory sound classification. Our evaluation was designed such that segmentation was performed consistently, and the cross-validation procedure was conducted in a manner ensuring that the test fold remained fully unseen during training.

The reviewers had already asked about all of these factors and more during the transparent review phase, and we had revised and updated everything accordingly.

In summary, we believe the issues raised by the commenter are primarily related to clarification and reporting detail rather than methodological inconsistency. We would be pleased to submit a short clarification note if required.
